# Supplementary material for: The Systems Biology Research Tool: evolvable open-source software
Source: BMC Syst Biol. 2008 Jun 29;2:55. doi: 10.1186/1752-0509-2-55 (PMC2446383; doi:10.1186/1752-0509-2-55)
Supplement: Additional file 1 — SBRT Archive. An archive of the current version of the Systems Biology Research Tool. [file 1752-0509-2-55-S1.zip › sbrt-1.4.0/doc/developers_guide/api/sbrt/kernel/fba/comp/package-frame.html]

sbrt.kernel.fba.comp


sbrt.kernel.fba.comp

Interfaces 
  
*CycleComputer*
  
*ExtremeCurrentComputer*
  
*FluxCvComputer*
  
*FluxIntervalComputer*
  
*FluxomeReducer*

Classes 
  
AbstractCvComputer
  
AbstractFluxIntervalComputer
  
CdHarFluxVectorComputer
  
FastMsFluxRangeComputer
  
FbaOptComputer
  
FbaSolver
  
FluxCapComputer
  
InitialPointComputer
  
MsFluxIntervalComputer
  
MsFluxomeReducer
  
ReverseRxnFluxIntervalComputer
  
RxnDeletionComputer
  
SimpleFluxIntervalComputer
  
SlpCycleComputer
  
SlpExCurrComputerV2
  
WwFluxomeReducer

Exceptions 
  
FbaOptException
  
FluxCapException
  
FluxRangeException
  
InvalidFluxVectorException
